# Is there any relationship between medication compliance and affective temperaments in patients with type 2 diabetes?

**DOI:** 10.1186/s40200-014-0096-z

**Published:** 2014-09-27

**Authors:** Alireza Shamsi, Fatemeh Khodaifar, Seyed Masoud Arzaghi, Farzaneh Sarvghadi, Arash Ghazi

**Affiliations:** Department of Psychiatry, Ali Ibnabitaleb hospital, Zahedan University of Medical Sciences, Zahedan, Iran; Department of Psychosomatic Medicine, Ayatollah Taleghani hospital, ShahidBeheshti University of Medical Sciences, Tehran, Iran; Endocrinology and Metabolism Research Center, Endocrinology and Metabolism Research Institute, Tehran University of Medical Sciences, Tehran, Iran; Endocrine Research Center, Research Institute for Endocrine Sciences, Shahid Beheshti University of Medical Sciences, Tehran, Iran; Wilderman Medicine Professional Corporation, Thornhill, Ontario Canada

**Keywords:** Medication compliance, Affective temperaments, Type 2 diabetes

## Abstract

**Background:**

Type 2 diabetes mellitus (DM) is the most common type of diabetes.The number of patients with this disease is expected torise in future. Given the increasing prevalence of diabetes, there is an urgent need for the treatment of diabetes and the associated complications. Glycemic control largely depends on compliance with medication therapies. In fact, the most common problem in patients with diabetes is lack of medication compliance. This study aimed to determine the relationship between affectivetemperaments and medication compliance in patients with type 2 diabetes.

**Methods:**

In this cross-sectional research, the study population consisted of all patients referring to the endocrinology clinic of Ayatollah Taleghani Hospital of Tehran in 2010 and 2011. Two hundreds and seven patients were selected, using available sampling method. In this study, we used Temperament Evaluation of Memphis, Pisa, Paris, and San Diego Auto questionnaire (TEMPS-A), a single-item scale of medication compliance, Beck Depression Inventory-II (BDI-II), and a researcher-made questionnaire to assess the patients’ demographic information. All participants completed the questionnaires related to affective temperaments, medication compliance, depression and demographic information. The obtained data were recorded on the prepared sheets.

**Results:**

Of 207 patients, 79 (38.2%) and 128 (61.8%) subjects were male and female, respectively. The mean and standard deviation of demographic data were calculated. In total, 13.5%, 19.3%, and 8.2%of the participants had mild, moderate, and severe depression, respectively. In this study, as the single-item rating scale indicated, medication compliance and non-compliance were reported in 75.4% and 24.6% of the patients, respectively. Among the demographic characteristics and clinical variables, frequency of patient referral and glycated hemoglobin level were predictors of medication compliance. Also, among affective temperaments, irritable temperament was a predictor of medication compliance.

**Conclusions:**

The obtained findings emphasize the importance of psychological factors such as personality characteristics in medication compliance of patients with diabetes. In case a patient obtains high scores in irritable temperament (which indicate poor medication compliance), he/she should follow special training programs to improve his/her medication compliance.

## Background

Diabetes mellitus (DM) is one of the most common chronic diseases, worldwide [1]. Diabetes prevalence has significantly increased due to the growing of obesity and increased life expectancy [2].

The prevalence of DM in Iran has been estimated at 4.67% among people older than 20 years of age [3]. According to World Health Organization (WHO), there are more than 2 millions patients with diabetes in Iran. This numberis expected to increase to 6.4 millions in 2030 [4].

Type 2 DM is the most common type of diabetes. This disease is a complex disorder which requires constant attention to diet, blood glucose monitoring, and medication consumption for glycemic control [5]. Given the increasing prevalence of diabetes, patients and healthcare systems need to spend significant amounts of money for the management and treatment of diabetes and its acute and chronic complications [3].

Glycemic control largely depends on compliance withmedication therapies. In fact, the most common problem in patients with diabetes is non-compliance with medications [4,6]. Medication non-compliance leads to the increased prevalence of diabetes-associated complications. It also reduces the patients’ quality of life and increases mortality and morbidity rates [3,7]. Moreover, patients’ refusal of treatments leads to an increase in costs, arising from disease complications [7]. Only 43% of patients with diabetes have glycated hemoglobin level below 7% [4,6].

So far, few studies have been conducted about the causes of non-compliance withtreatments. Some of these studies have examined the relationship between psychosocial factors and medication compliance. These studies have shown that some of these factors have significant effects on the prognosis of diabetes [8].

Temperament, character and personality are terms used to distinguished enduring personal characteristics. Temperament is closer to “humoral theory” and represents the biological core of personality with genetic and constitutional endowment. Character is more linked to nurture, comprising acquired competencies during development, namely interpersonal development. Personality covers a wider scope bridging inborn temperamental and learned characterologic features associated with psychological and behavioral traits [9].

Given the limited number of studies examining the relationship between temperaments and medication compliance in Iranian population, this study aimed to determine the relationship between affective temperaments and medication compliance in patients with type 2 diabetes.

## Methods

The population of this cross-sectional study consisted of all patients referring to the endocrinology clinic of Ayatollah Taleghani Hospital of Tehran in 2010 and 2011. By using available sampling method, 215 patients were included in the study.

First, the patients who met the inclusion criteria were selected. Of 215 participants, eight people with incomplete questionnaires were excluded from the study. At the end, 207 subjects were enrolled in the study.

### Inclusion and exclusion criteria

The inclusion criteria were as follows: 1) ≥18 years of age; 2) having type 2 DM; 3) proficiency in reading and writing Farsi; 4) providing informed consents; and 5) having been diagnosed with diabetes for at least one year.

Patients with severe cognitive problems were excluded from the study. Lack of patient’s consent to participate in the study was another exclusion criterion.

### Study instruments

#### TEMPS-A

To assess the patients’ affective temperaments, we used Temperament Evaluation of Memphis, Pisa, Paris, and San Diego Auto questionnaire (TEMPS-A). This questionnaire was designed by Akiskal HS et al. to assess five affective temperaments including depressive, cyclothymic, hyperthymic, irritable, and anxious temperaments [10]. This scale consists of 110 items for women and 109 items for men. Items 1–21, 22–42, 43–63, 64–84, and 85–110 of this questionnaire assess the depressive, cyclothymic, hyperthymic, irritable, and anxious temperaments, respectively. All yes/no items were answered by the participants. TEMPS-A was validated in Iran by Meshkat Razavi G and Sardarpur Gudarzi S on 683 university students. Cronbach α values was 0.88 (depressive), 0.76 (cyclothymic), 0.90 (hyperthymic), 0.76 (irritable), and 0.78 (anxious) [11].

### Single-item scale of medication compliance

In this study, we used a single-item rating scale to assess medication compliance. The patients were asked to answer the following question: “To what degree have you complied with prescribed treatments over the past month?” The answers were rated on a 5-point likert scale, ranging from 1 to 5 (1: never, 2: less than half the time, 3: about half the time, 4: more than half the time, and 5: always). Scores 3 and less than 3 indicated medication non-compliance, and scores higher than 3 indicated medication compliance. In similar studies, this scale was used for measuring medication compliance in patients with diabetes, hypertension, and heart conditions [9,10].

### Beck Depression Inventory-II (BDI-II)

Beck Depression Inventory was designed by Aaron T. Beck. This instrument is a 21-item, multiple-choice, self-report inventory, which is used for measuring the severity of depression among patients. It consists of items related to depression symptoms (hopelessness and irritability), cognition (guilt or feelings of being punished), and physical symptoms (fatigue, weight loss, and lack of interest in sex). BDI-II is a revision of BDI, designed in 1996.

### Researcher-made questionnaire

This questionnaire was used to gather demographic information. The demographic data consisted of age, sex, marital status, and level of education. The information related to the time of diabetes diagnosis (and physical conditions) and the type of psychiatric medications (used by patients) was also gathered in this questionnaire.

### Ethical considerations

For ethical considerations, the consents of the authorities of the hospital, endocrinology clinic, and psychiatry department were obtained (Shahid Beheshti University of Medical Sciences). Also, all patients were given assurance about the confidentiality of the data; informed consents were obtained from the participants.

### Data analysis

All participants in this study completed the questionnaire related to affective temperaments, medication compliance, and depression; they also filled the researcher-made questionnaire. The information related to the level of glycated hemoglobin and the frequency of patient referral within the last year was extracted from patients’ records. Afterwards, the obtained data were recorded on the prepared sheets.

At first, the collected data were entered to SPSS version 19. The mean of quantitative data (age, disease duration, frequency of patient referral, level of glycated hemoglobin, and temperament scores) and the frequency of qualitative data (sex, marital status, and drug compliance) were calculated. The distribution of samples and comparison of quantitative variables were evaluated by Kolmogorov-Smirnov test, t-test, and Man-Whitney test. The comparison of qualitative variables was performed by Chi-square test. Also, logistic regression was used for modeling the relationship between medication compliance and the associated variables. Also, goodness of fit was tested by Chi-square.

## Results

In this study, 207 patients, diagnosed with type 2 diabetes, were assessed. Overall, 79 (38.2%) and 128 (61.8%) patients were male and female, respectively. The subjects were within the age range of 18–81 years; also, the mean and standard deviation of age were 48.4 ± 14.3 years. Evaluation of patients’ marital status showed that 14%, 72.5%, 6.8%, and 6.8% of the subjects were single, married, divorced, and widowed, respectively.

The evaluation of the subjects’ education level showed that 26.6% of the participants had less than high school education. Also, 31.9%, 12.1%, 17.9%, and 11.6% of the subjects had high-school diploma, associate degree, bachelor’s degree, and higher than bachelor’s degree, respectively.

The mean and SD of the time of diagnosis, the frequency of patient referral, and the last HbA1C test result were 7.9 ± 6.8, 3.6 ± 18, and 8.04 ± 1.9, respectively. In addition, 13.5%, 19.3%, and 8.2% of the subjects had mild, moderate, and severe depression, respectively. Eighty seven percent of patients did not receive psychiatric medications and the rest were treated by antidepressants.

As the evaluation by single-item rating scale indicated, medication compliance was reported in 75.4% of the patients; also, non-compliance with medication was reported in 24.6% of the subjects. Comparisons between patients’ demographic characteristics and medication compliance/non-compliance are shown in Tables 1 and 2.

**Table 1:** **Comparison between the demographic characteristics (time of diagnosis, frequency of patient referral, and last HbA1C results) of patients with and without medication compliance**

	**Total**	**Medication compliance**	**Medication non-compliance**	**P-value**	**Test**
**Age**	48.4 ± 14.3	47.5 ± 14.5	51.3 ± 13.3	0.093	t-student
**Time of diagnosis**	7.9 ± 6.8	7.3 ± 6.3	9.9 ± 7.9	0.033	Mann–Whitney
**Frequency of patient referral**	3.6 ± 1.8	4.03 ± 1.7	2.1 ± 1.1	0.0001	Mann–Whitney
**Last HbA1C results**	8.04 ± 1.9	7.4 ± 1.5	9.9 ± 1.8	0.0001	Mann–Whitney

**Table 2:** **Comparison of demographic characteristics (sex, marital status, education level, and depression) of patients with and without medication compliance**

		**Total**	**Medication compliance**	**Medication non-compliance**	**P-value**	**Test**
**Sex**	Male	38.2%	38.5%	37.3%	0.878	Chi-square
	Female	61.8%	61.5%	62.7%		
**Marital status**	Single	14%	17.3%	3.9%	0.006	Chi-square
	Married	72.5%	73.1%	70.6%		
	Divorced	6.8%	4.5%	13.7%		
	Widowed	6.8%	5.1%	11.8%		
**Education level**	Less than high school education	26.6%	23.7%	35.5%	0.318	Chi-square
	High school	31.9%	30.8%	35.5%		
	Associate degree	12.1%	12.8%	9.8%		
	Bachelor’s degree	17.9%	19.9%	11.8%		
	Higher than bachelor’s degree	11.6%	12.8%	7.8%		
**Depression**	Minor	58.9%	64.7%	41.2%	0.019	Chi-square
	Mild	13.5%	12.8%	15.7%		
	Moderate	19.3%	16%	29.4%		
	Severe	8.2%	6.4%	13.7%		

At first, the relationships between affective temperaments, BDI-II score, and medication compliance were examined by t-test and Mann–Whitney test. According to the obtained results, depressive, cyclothymic, irritable, and anxious temperaments and BDI-II score were inversely related with medication compliance. The comparison between affective temperaments and BDI-II score, based on drug compliance, is shown in Table 3.

**Table 3:** **Comparison between emotional temperaments (depressive, cyclothymic, irritable, and anxious) and BDI-II score, based on medication compliance and non-compliance**

	**Medication compliance**	**Medication non-compliance**	**P-value**	**Test**
Depressive temperament	1.4 ± 0.2	1.5 ± 0.2	0.010	Mann–Whitney
Cyclothymic temperament	1.3 ± 0.3	1.4 ± 0.2	0.009	Mann–Whitney
Hyperthymic temperament	1.5 ± 0.3	1.5 ± 0.2	0.612	t-test
Irritable temperament	1.1 ± 0.2	1.3 ± 0.2	0.0001	Mann–Whitney
Anxious temperament	1.3 ± 0.2	1.4 ± 0.2	0.048	Mann–Whitney
Depression inventory score	12.4 ± 9.3	17.8 ± 11.0	0.001	Mann–Whitney

Then we used logistic regression for modeling the relationship between medication compliance and the related variables. According to the obtained results of this study, among demographic characteristics and clinical variables, the frequency of patient referral and glycated hemoglobin level were predictors of medication compliance. Also, among affective temperaments, only irritable temperament was the predictor of medication compliance after using logistic regression. The results of logistic regression are shown in Tables 4, 5, and 6.

**Table 4:** **The relationship between demographic data, clinical variables, and medication compliance**

**Model 1**	**Chi-square (sig)**	**Overall correct (%)**	**Odds ratio (CI)**
**Best fit line**	10.551 (0.228)	86.5%	-
**Age**	-	-	1.4 (1.003-1.07)
**Frequency of patient referral**	-	-	2.25 (1.5-3.2)
**Glycated hemoglobin**	-	-	0.393 (0.28-0.54)

**Table 5:** **The relationship between affective temperaments and medication compliance**

**Model 2**	**Chi-square (sig)**	**Overall correct (%)**	**Odds ratio (CI)**
**Best fit line**	8.756 (0.271)	77.7%	-
**Irritable temperament**	-	-	0.028 (0.005-0.166)

**Table 6:** **The relationship between demographic characteristics, affective temperaments, and medication compliance**

**Model 3**	**Chi-square (sig)**	**Overall correct (%)**	**Odds ratio (CI)**
**Best fit line**	9.12 (0.332)	87.4%	-
**Frequency of patient referral**	-	-	1.94 (1.37-2.74)
**Glycated hemoglobin**	-	-	0.41 (0.29-0.56)
**Irritable temperament**	-	-	0.02 (0.001-0.332)

## Discussion

According to the obtained results of this study, medication compliance and non-compliance were reported in 75.4% and 24.6% of the patients, respectively. However, the prevalence of medication non-compliance in other studies varied from 36% to 93% [12,13]. This discrepancy may be related to differences in study design, demographic characteristics, and patients’ education level. Also, high medication compliance in our study may be rooted in the bias in the selection of patients.

Similar to previous research [14,15], our study showed that the rate of medication compliance decreased with increasing age. Also, in consistence with the results of previous studies, it was revealed that the level of glycated hemoglobin was lower in patients with medication compliance and the frequency of patient referral was higher in patients with medication compliance [16]. In addition, similar to the present study, previous research showed no relationship between gender and medication compliance [17].

In our study, there was no relationship between the time of diagnosis and medication compliance; this finding is confirmed by the results of other studies [18]. Also, there was no association between marital status and medication compliance. However, in some studies, medication compliance was higher in married individuals [19,20].

We did not find a correlation between the level of education and medication compliance; this finding was inconsistent with those of other studies [7], which may be due to the classification of education variable in our study. According to the obtained results, depressive, cyclothymic, irritable, and anxious temperaments were inversely related with medication compliance. However, after using logistic regression there was only an inverse relationship between irritable temperament and medication compliance and there was no relationship between other affective temperaments and medication compliance.

In consistence with our study, the study by Gois et al. aimed to determine the relationship between depressive and anxious temperaments and medication compliance. The results of their study showed that high scores of depressive and anxious subscales were correlated with medication non-compliance [9]. Their findings are in part in line with our study because depressive, anxious, and irritable temperaments are in the same cluster and in all of them there is a strong involvement of the central serotonergic system [21]. However, the study by Hall et al. showed that anxious temperament was not associated with medication compliance [22].

Yoda et al. performed a study to examine the relationship between personality traits and level of glycated hemoglobin. They showed a positive significant relationship between glycated hemoglobin level and anxious temperament [10]; the results of this study were confirmed by those obtained by Gois and colleagues [9].

In addition, very few studies have been conducted about the relationship between medication compliance and affective temperaments (except for depressive and anxious temperaments) in patients with type 2 diabetes. This may reflect the lack of attention to the relationship between medication compliance and personality characteristics. However, given the differences in personality characteristics of individuals, people need special training for the management of diseases. Therefore, it is recommended that a training program be implemented for patients, who are newly diagnosed with diabetes, based on their personality characteristics [7,8,23].

The obtained results emphasize the importance of psychological factors such as personality characteristics in medication compliance of patients with diabetes. We should pay attention to poor medication compliance; lack of glycemic control, and psychological problems of patients with diabetes after diabetes diagnosis; if necessary, patients should be referred for psychiatric counseling. In case a patient obtains high scores in irritable temperament (which indicate poor medication compliance), he/she should follow special training programs to improve his/her medication compliance.

### Limitations and strengths

In this study, a major weakness was the limited selection of patients, who were recruited from Ayatollah Taleghani Hospital. In addition, only subjects, who could read and write, were included in the study, which limited the obtained results. Also, this research was across-sectional study, thus, the obtained findings had lower power.

On the positive side, the sample size of the current study was larger than that of other similar researches. In addition, the correlation between medication compliance and all affective temperaments was assessed in the present study. In fact, no similar studies have been conducted in Iranian population.
